# Integrating transcriptome and metabolomics revealed the key metabolic pathway response of *Amaranthus retroflexus L*. to the resistance to fomesafen

**DOI:** 10.1371/journal.pone.0312198

**Published:** 2025-02-13

**Authors:** Weifeng Song, Qinghui Wei, Zhenghao Shi, Yaqing Pan, Zhiyong Li, Fangyuan Wang

**Affiliations:** Institute of Plant Protection, Heilongjiang Academy of Agricultural Sciences, Harbin, Heilongjiang, P. R. China; Central University of Haryana School of Life Sciences, INDIA

## Abstract

**Background:**

*Amaranthus retroflexus L*. is one of the main broad-leaved weeds in soybean fields in Heilongjiang Province and is an important factor affecting soybean yield. It is becoming increasingly resistant to herbicides. However, studies on the transcriptome level and the molecular mechanism of secondary metabolite accumulation of resistant varieties of *Amaranthus retroflexus L*. have not been reported. Therefore, comprehensive analysis of transcriptome and metabolome is needed to determine the key metabolic pathways and key genes of *Amaranthus retroflexus L*.

**Results:**

The biosynthetic pathway of resistance to *Amaranthus retroflexus L*. was studied by transcriptome and metabolome analysis. Transcriptome analysis showed that in the three comparison groups, compared with untreated (CK) group, there were 979 Differentially expressed genes (DEGs) in resistant (RY) group and 15731 DEGs in sensitive (SY) group; The RY group had 13822 DEGs compared to the SY group. Fluorescent quantitative PCR detection found that two gene tables related to Cytochrome P450 Monooxygenase (P450), Glutathione S-transferase (GST) and other enzyme systems such as peroxidase (POD), polyphenol oxidase (PPO), Catalase (CAT) and Superoxide dismutase (SOD) were significantly reached. Using Venn analysis for metabolomics analysis (VIP>1 and P<0.05), 239 Differentially expressed metabolites (DEMs) were selected. There are 15 common DEMs in the three control groups, and 8 unique DEMs in the RY group. This study detected 76 cases of DEMs and 139 cases of DEMs in the CK, RY, and SY control groups, respectively. More metabolites were detected in the CK and SY control groups. This viewpoint provides evidence for the genetic and metabolic differences between resistance and sensitivity in *Amaranthus retroflexus L.*. The KEGG in the RY vs SY group is mainly enriched in cysteine and methlonine metabololism, glycine, serine and threonine metabololism, aminoacyl-tRNA biosynthesis, biosynthesis of variant plant secondary metabololites, biosynthesis of amino acids, arginine and proline metabololism, biosynthesis of cofactors. Therefore, the resistance mechanism of *Amaranthus retroflexus L*. may be mainly generated by the metabolic pathway mechanism of amino acids.

**Conclusion:**

In this study, DEGs and DEMs were identified by de novo Transcriptome assembly and metabonomic analysis, and an important metabolic pathway of resistance was found. It was found that the resistance mechanism of *Amaranthus retroflexus L*. might be mainly produced by amino acid metabolic pathway. This discovery laid the foundation for further research on the molecular mechanism and functional characteristics of the resistance of *Amaranthus retroflexus L.*.

## 1. Introduction

*Amaranthus retroflexus L*., also known as Xifenggu and Wild Rice, it is currently one of the main broad-leaved weeds in soybean fields in Heilongjiang Province [1,2]. When the *Amaranthus retroflexus L*. invades farmland, it occupies farmland space through the higher part of the ground, competing with crops for light, water, fertilizer, thereby reducing crop yield [3]. Fomesafen is a diphenyl ether herbicide that acts on protoporphyrin peroxidase (PPO). Since its widespread use in the 1990s, it has been the main herbicide for controlling broad-leaved weeds in soybean seedling stem and leaf treatment due to its good weed control effect, wide weed control spectrum, and strong selectivity [4]. But with long-term and large-scale use, the resistance of *Amaranthus retroflexus L*. to fomesafen is becoming increasingly serious [5]. Resistant weeds have always been a key factor affecting soybean yield. If the weed control effect is not good, it will lead to serious reduction or even crop failure of soybeans [6]. Therefore, elucidating the resistance mechanism of *Amaranthus retroflexus L*. to fomesafen provides a theoretical basis for precise control of weeds in soybean fields, which has important theoretical and practical significance.

With the development of transcriptome sequencing (RNA-Seq) and metabonomics, substantial progress has been made in the molecular mechanisms of weed resistance [7,8]. For example, studies have found that two P450 genes (RrCYP704C1 and RrCYP709B1) and one dioxygenase gene (Rr2ODD1) in wild radish are genes related to the metabolic resistance of hydroxyphenylpyruvate dioxygenase (HPPD) herbicides. In another study, iTRAQ proteomics quantitative technology was used to study the non-resistant and resistant Kanmai Niang, a weed. The results of differential protein analysis showed that herbicides would cause damage to weed plants in photosynthesis, redox balance and other processes. In contrast, herbicides in resistant plants are rapidly degraded, thereby reducing the chemical damage caused by herbicides to their metabolic pathways such as photosynthesis. Further research results indicate that esterase, GST, and glucosyltransferase can serve as potential markers to quickly indicate the metabolic resistance of weed plants [9–11].

Previous studies have investigated the plant toxicity mechanism of Sterigmatocystin (STE) through the metabolomics of *Amaranthus retroflexus L.*. 140 and 113 different metabolites were detected in leaves and stems, respectively, with significant interference from amino acids, lipids, and phenolic compounds. Valine, leucine, isoleucine, and lysine biosynthesis were affected by STE [12]. Arg-128-Gly substitution is the main reason for *Amaranthus retroflexus L*. to develop resistance to fomesafen. This is the first report on the target site mechanism of resistance of Bacillus retroflexus to herbicides that inhibit protophyrinogen oxidase [13]. However, the mechanism of the response of *Amaranthus retroflexus L*. to fomesafen resistance is currently not clear, and research is limited to mutation mutations and some target genes [14]. There is no report on the mechanism of comprehensive research on transcriptome and metabolome, and at present, in Heilongjiang Province, the field application of fomesafen in Heilongjiang Province has failed to control the *Amaranthus retroflexus L*. population [13]. Therefore, it is necessary to further integrate transcriptome and metabolomics to reveal the key metabolic pathway response of *Amaranthus retroflexus L*. to fomesafen resistance, so as to provide molecular basis for *Amaranthus retroflexus L*. after leaf treatment and achieve the purpose of improving soybean yield in Heilongjiang.

Previous studies have found that *Amaranthus retroflexus L*. undergoes changes in metabolic levels after drug treatment [12], and the expression levels of some target genes in *Amaranthus retroflexus L*. after treatment with fomesafen [14]. Metabolites are products of gene function, therefore, the transcriptional and metabolic levels of *Amaranthus retroflexus L*. may change after treatment with fomesafen, leading to resistance. In this study, transcriptome and metabonomics analysis were carried out on the role of fomesafen, and the response mechanism of the key metabolic pathway for drug resistance to fomesafen was discussed. Provide theoretical and practical basis for the prevention and control of drug-resistant *Amaranthus retroflexus L.*.

## 2. Materials and methods

### 2.1 Reagents and weeds

250g/L fomesafen ether aqueous solution (commercially available by Liansongliao Chemical Co., Ltd.). In 2017 and 2018, *Amaranthus retroflexus L*. seeds were collected from soybean fields in Bei’an City, Heilongjiang Province, and dried and stored in a refrigerator at (4 ± 1)°C for future use. In short, a population of fomesafen resistant *Amaranthus retroflexus L*. was collected from a soybean field, where fomesafen was not controlled at the recommended field dose (275 g ai ha-1). Collect sensitive populations of *Amaranthus retroflexus L*. seeds from wasteland and randomly divide them into two groups: one group was not treated with fomesafen, and the other group was treated with fomesafen. The whole plant bioassay method found that after treatment with the recommended field application amount of fomesafen (275 g ai ha-1), the growth of the sensitive population of *Amaranthus retroflexus L*. was completely inhibited, while the growth of the resistant population of *Amaranthus retroflexus L*. was not completely inhibited [13].

### 2.2 Transcriptome analysis

Novaseq 6000 (Illumina) was used for transcriptome sequencingTake 0.5 g of leaf tissue (3 replicates per group) from the sensitive (SY) and resistant (RY) and untreated (CK) groups treated with fomesafen, and freeze in liquid nitrogen. RNA was extracted from leaf tissue samples using TRIzol reagent (Invitrogen, USA) and purifies it with plant RNA purification reagent (Invitrogen company), Then, the cleaved RNA fragments were reverse-transcribed to create the final cDNA library following the protocol for the mRNA Seq sample preparation kit (Illumina, San Diego, CA, USA). The average insert size for the paired-end libraries was 300 bp (±50 bp). Differentially expressed genes (DEGs) were identified between the CK and fomesafen treatment groups using DEGseq2. The function annotation of DEGs was carried out by the free online platform of Majorbio.com (www.Majorbio.com) for Swiss Prot annotation, homologous protein cluster (COG), Gene Ontology (GO) classification and Kyoto Gene and Genome Encyclopedia (KEGG) database [15,16].

### 2.3 Quantitative real-time PCR analysis of genes

**Fig 1:**
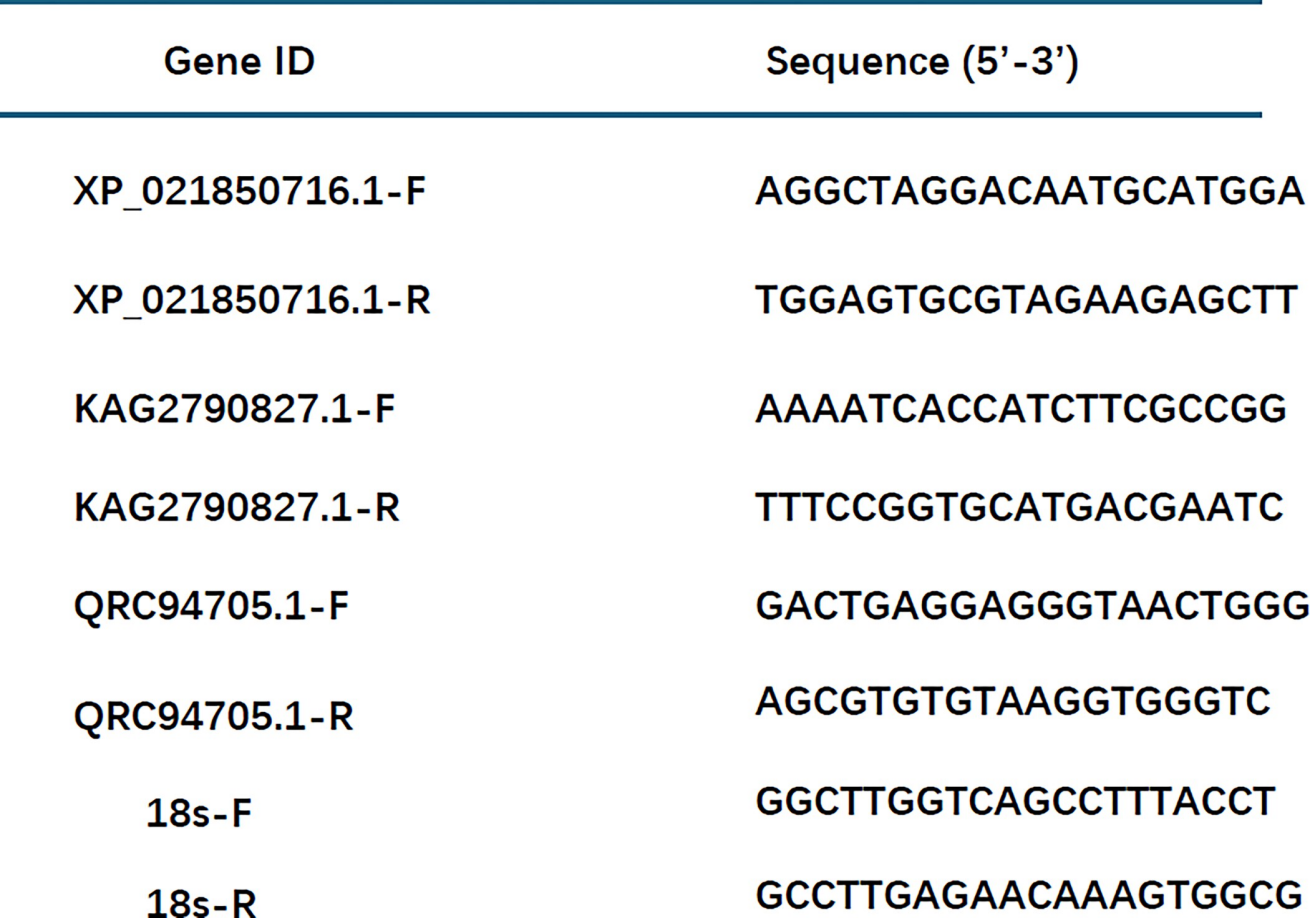
The primer design sequence. The primer design sequence table for three genes is shown in Fig 1. F: Forward primer, R: Reversed primer.

The non-target resistance mechanism of common weeds was metabolic resistance, which was generally caused by the enhancement of cytochrome P450 Monooxygenase (P450s), glutathione S-transferase (GST) and other enzyme systems, such as peroxidase (POD), polyphenol oxidase (PPO), hydrogen oxide (CAT) and superoxide dismutase (SOD). In order to verify the data of the transcriptome and the expression of genes related to resistant enzymes, three single genes were selected for real-time quantitative PCR (qRT-PCR). Primer Premier 5.0 was used to design primers for each single gene. 18s F1 and 18s R1 were used as internal reference genes. The primer design sequence table for three genes is shown in Fig 1. F: Forward primer, R: Reversed primer. The 2-ΔΔCt method was used to determine the relative expression of each gene in different samples [17].

### 2.4 Metabolomics analysis

Extract leaf tissue from the fomesafen treated group and the untreated CK group (3 replicates per group) at a quantity of approximately 1 g (3 replicates per group), weigh, and freeze in liquid nitrogen. After natural air drying, grind the leaf sample into powder, extract and analyze metabolites. 60 mg of ground powder was extracted by ultrasound with 0.6 ml methanol/water (7:3, v/v) for 30 minutes, then incubated at -20°C for 20 minutes. Centrifuge the extract at 14000 rpm for 10 minutes at 4°C. The supernatant was measured using the Waters VION IMS Q-TOF mass spectrometer (Waters Corporation, Milford, MA, USA) platform equipped with an electric spray interface, as described elsewhere. Differentially expressed metabolites (DEMs) between CK and fomesafen treatment groups were identified using Orthogonal Partial Least Squares Discriminant Analysis (OPLS-DA), and the OPLS-DA model was validated through permutation testing [18].

### 2.5 Correlation analysis between transcriptome and metabolome

Used Spearman correlation coefficient test to calculate the correlation coefficient between differential DEGs and differential DAMs. Select correlation coefficient ≥ 0.7 and P-value ≤ 0.5. Correlation maps were used to identify potential key genes and metabolites [19].

### 2.6 Statistical analysis

DEGs were identified using the DEGseq2 package. P value < 0.05 and |log2foldchange| > 1.0 was set as the threshold for significantly differential expression. GO enrichment, COG functional analysis and KEGG pathway enrichment analysis of DEGs were respectively performed using R based on the hypergeometric distribution. Multivariate statistical analysis used OPLS-DA to distinguish the overall differences of metabolic profiles between groups and find the different metabolites between groups. VIP>1 and P < 0.05 were considered statistically significant. The spearman analysis was used to assess the relationship between genes and metabolites. correlation coefficient ≥ 0.7 and p value ≤ 0.5 were considered statistically significant [20].

## 3 Results

### 3.1 Transcriptome analysis

#### 3.1.1 Transcriptome sequencing and functional annotation

**Fig 2:**
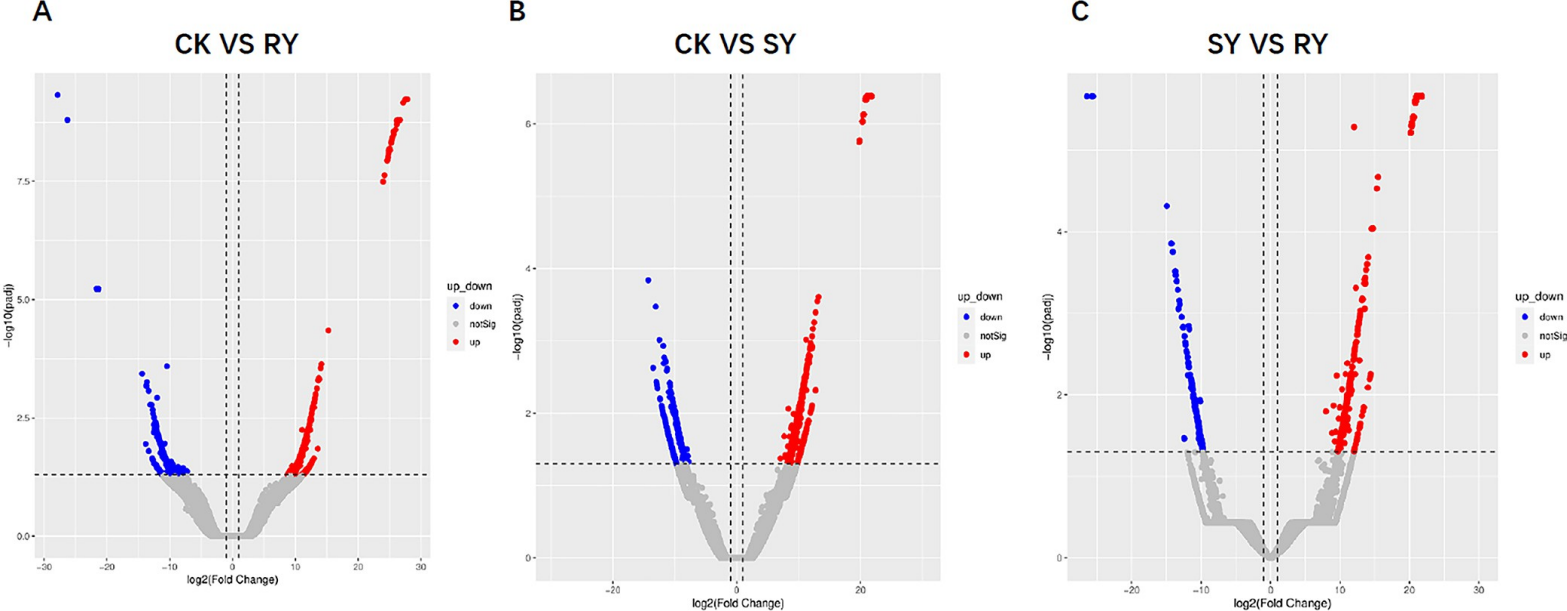
Three sets of volcanic maps compared to each other. (A) The volcanic map of CK VS RY shows that red represents significantly upregulated genes, blue represents significantly downregulated genes, and gray represents insignificant gene expression; (B) The volcanic map of CK VS SY shows that red represents significantly upregulated genes, blue represents significantly downregulated genes, and gray represents insignificant gene expression; (C) The volcanic map of SY VS RY shows that red represents significantly upregulated genes, blue represents significantly downregulated genes, and gray represents insignificant gene expression.

We obtained clean data from a total of 9 samples for three groups (CK group, RY group, and SY group). All Q30 values are greater than 80%, indicating the high reliability of the sequencing results. After obtaining the spliced transcript, functional annotation is required to obtain the functional information of the gene. To obtain comprehensive gene function information, seven databases of gene function annotations were conducted on the unigene sequence, including: Nr, Nt, KOG, Swiss prot, Uniprot. Detailed annotation information for Unigenes from Non redundant (NR), GO, KEGG, and Non supervised Orthologous Groups (NOG) databases is displayed. This study analyzed the gene expression of each fomesafen group. Among them, the differential expression of genes in three different treatment groups is shown in the volcanic map (Fig 2A–2C).

#### 3.1.2 Identification of transcriptome DEGs

**Fig 3:**
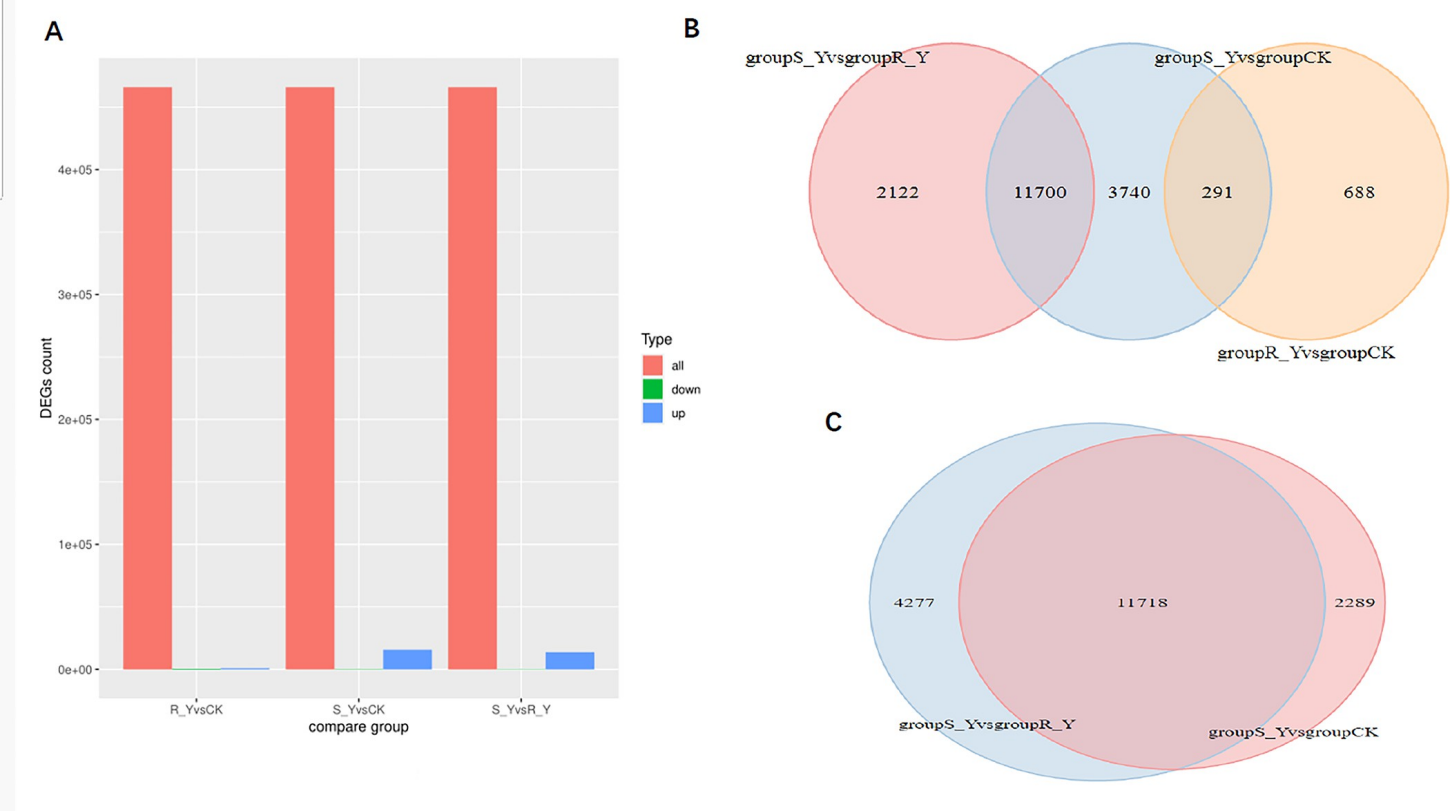
Grouping display of differentially expressed genes at transcriptional levels. (A) Red represents the number of detected genes, blue represents significantly upregulated genes, and green represents significantly downregulated genes; (B) Venn plot shows significant overlapping and non-overlapping genes among the three groups; (C) Venn plot shows significant overlapping and non-overlapping genes between SY group, CK group, and RY group.

The distribution of DEGs in different treatment groups is shown in (Fig 3A). This study compared the up-regulated and down-regulated DEGs in the CK and fomesafen treatment groups. We found that the RY vs CK group had the least number of differentially expressed genes, so the RY group may have a phenotype closer to the CK group compared to the SY group (which was successfully verified by subsequent metabolomics analysis), while the RY vs SY group showed significant differences. The Venn plot results show that among the three comparison groups, compared with the CK group, the RY group has 979 DEGs, the SY group has 15731 DEGs, and the RY vs SY group has 13822 DEGs (Fig 3B). In the comparison group between the CK and SY groups, the highest number of DEGs were detected, followed by the DEGs detected by the RY and SY groups. The CK and RY groups had the lowest number of DEGs detected. In addition, there were a large number of overlapping DEGs between the RY vs SY and CK vs SY groups, with overlapping genes accounting for more than 70% of the entire DEGs (Fig 3C), indicating that the resistant variety of *Amaranthus retroflexus L*. has a significant resistance effect on fomesafen. To prove the relevant results, We validated it through metabolomics. A study has reported that after treatment with fluconazole, the relevant indicators of the resistant population (RY) of the same *Amaranthus retroflexus L*. leaf age are less affected than those of the sensitive population (SY), and the recovery speed is faster [21]. We verified this at the gene Transcriptome level.

#### 3.1.3 Quantitative real-time PCR analysis of genes

**Fig 4:**
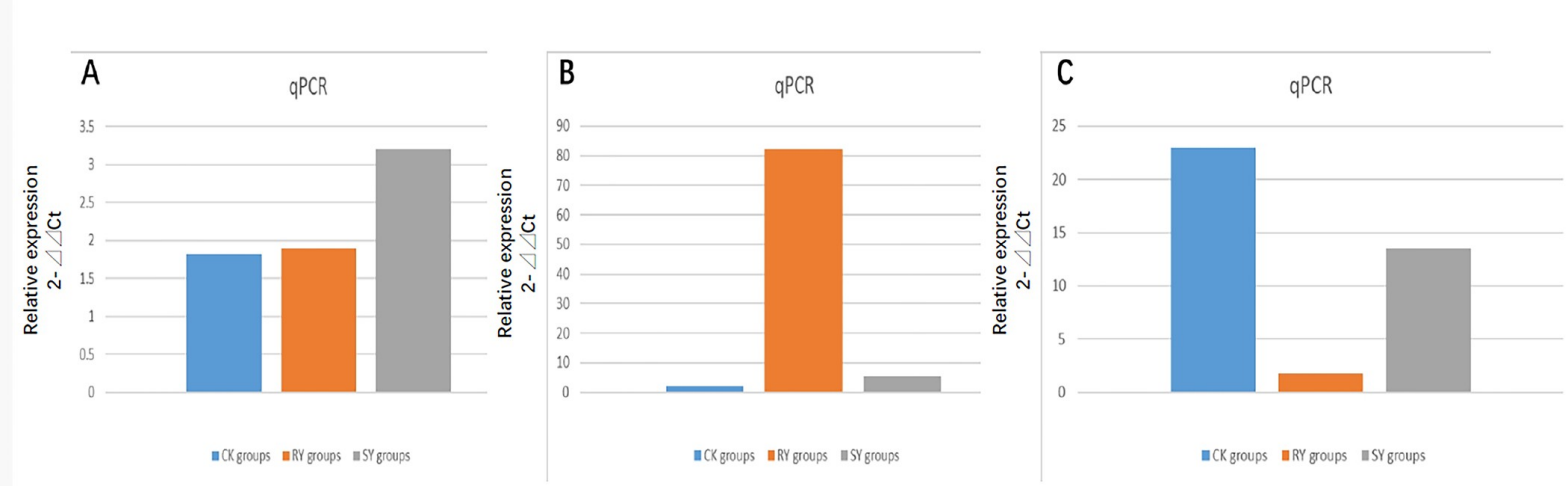
Quantitative real-time PCR analysis of genes. (A) Differential expression profile of XP_021850716.1 gene in CK, RY, and SY groups; (B) Differential expression profile of KAG2790827.1 gene in CK, RY, and SY groups; (C) Differential expression profile of QRC94705.1 gene in CK, RY, and SY groups.

The expression levels of three genes related to *Amaranthus retroflexus L*. resistance enzymes were verified between the three groups through RT-PCR (Gene ID sourced from NR database). It was found that the XP_021850716.1 gene showed no significant change in the RY group compared to the CK group, but significantly increased in the SY group (Fig 4A); Compared with the CK and SY groups, the expression level of KAG2790827.1 gene was significantly increased in the RY group (Fig 4B), while the expression level of QRC94705.1 gene was significantly reduced in the RY group (Fig 4C). Therefore, the relevant results indicate that the resistance of *Amaranthus retroflexus L*. is reflected at the transcriptional level, providing a molecular basis for the management of *Amaranthus retroflexus L*. resistant varieties.

### 3.2 Metabolomics analysis

#### 3.2.1 The effect of fomesafen on the metabolism of retroflex amaranth leaves

**Fig 5:**
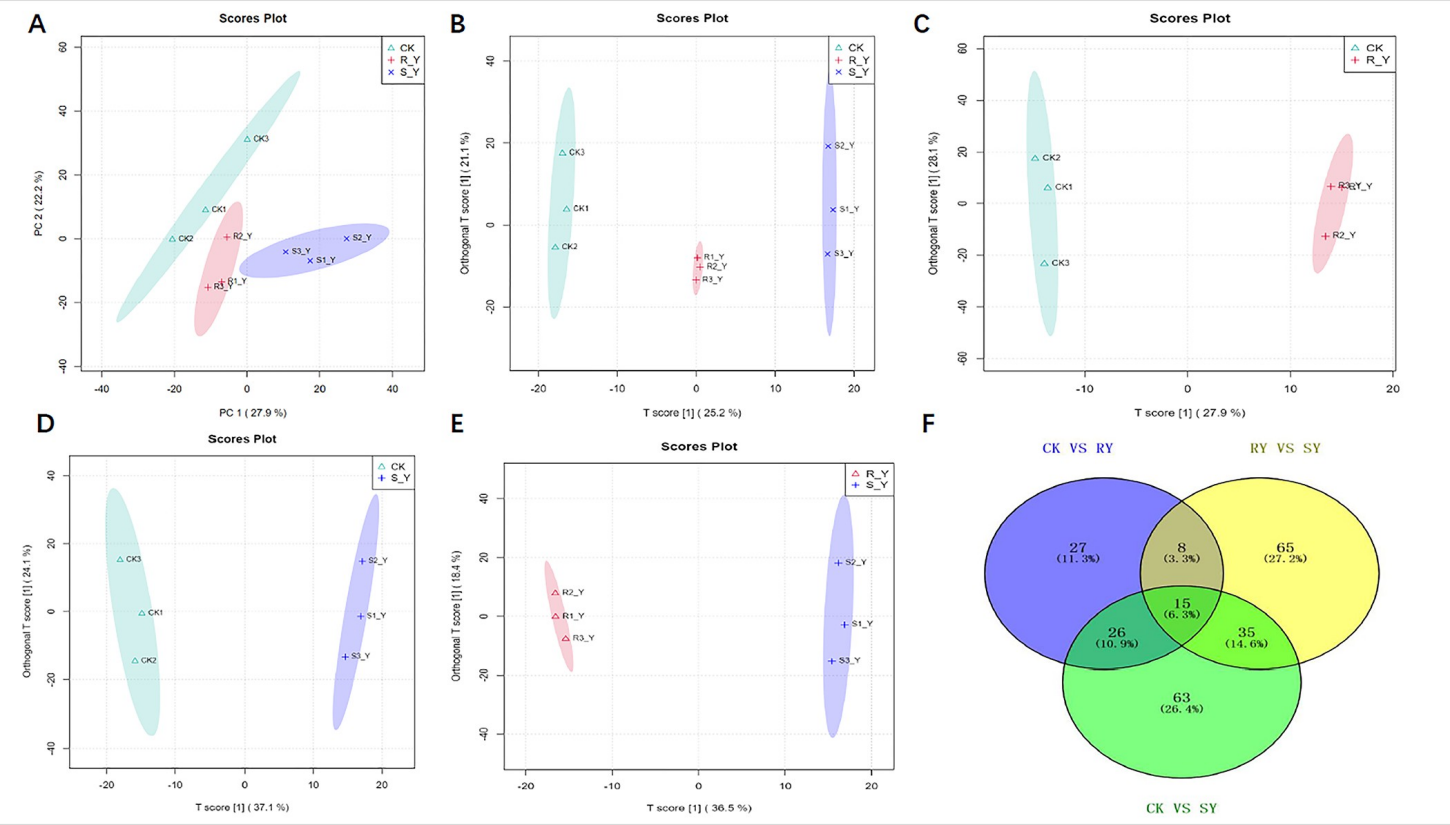
Display of metabolic differences between the three groups in metabolomics. (A) PCA diagram of metabolites between three groups in metabolomics; (B) OPLS-DA diagram of metabolites between three groups in metabolomics; (C) OPLS-DA diagram of metabolites between CK group and RY group in metabolomics; (D) OPLS-DA diagram of metabolites between CK group and SY group in metabolomics; (D) OPLS-DA diagram of metabolites between RY group and SY group in metabolomics; (D) Venn plot shows significant overlapping and non-overlapping genes between SY group, CK group, and RY group.

We conducted multivariate PCA analysis and OPLS-DA analysis on the clustering information of three groups and validated the OPLS-DA model through permutation testing. The results showed a significant partition between the control group and the fomesafen treated group (Fig 5A and 5B), and it was found that the metabolic phenotype of the RY group was closer to that of the CK group, indicating that the RY group was not sensitive to the effects of fomesafen due to the presence of resistance mechanisms. In OPLS-DA analysis, significant differences were also observed between different fomesafen treatment groups (Fig 5C–5E). The results showed that there was a significant difference in metabolites between the RY group and the SY group under fomesafen treatment. In addition, this study selected 239 DEMs through Venn analysis (VIP>1 and P<0.05). There are 15 common DEMs among the three control groups, and 8 unique DEMs among the RY group (Fig 5F). This study detected 76 DEMs and 139 DEMs in the CK, RY, and SY comparison groups, respectively. More metabolites were detected in the CK vs. SY comparison group (Fig 5F). Therefore, the RY group is closer to the metabolic phenotype of the CK group, demonstrating resistance to fomesafen.

#### 3.2.2 KEGG pathway enrichment analysis

**Fig 6:**
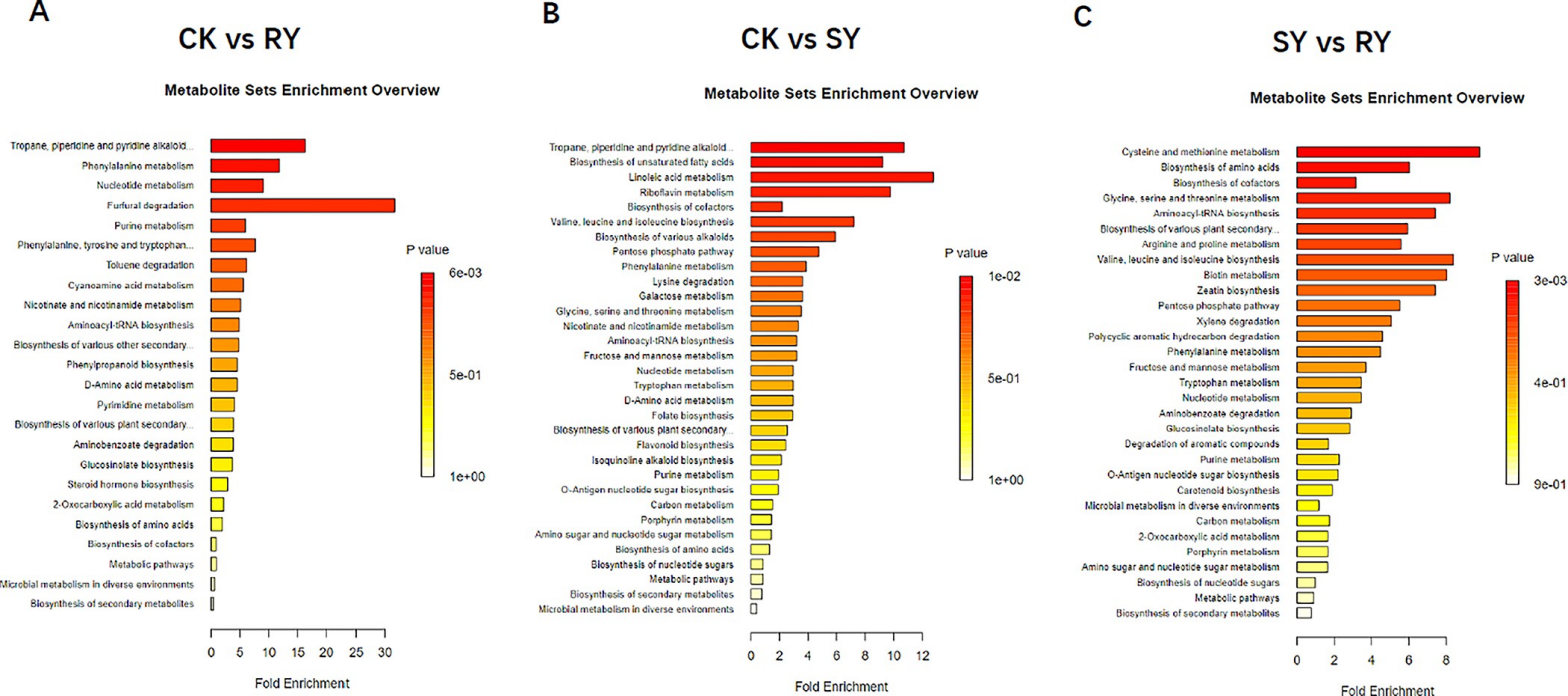
KEGG enrichment pathway map of differential metabolites. (A) KEGG enrichment pathway diagram of differential metabolites between CK and RY groups (P<0.05); (B) KEGG enrichment pathway diagram of differential metabolites between CK and SY groups (P<0.05); (C) KEGG enrichment pathway diagram of differential metabolites between SY and RY groups (P<0.05).

In order to directly analyze the differences in metabolic pathways between different control groups, the key metabolic pathway of the response of *Amaranthus retroflexus L*. to fomesafen was further determined. This study selected the top 20 metabolic pathways with differential enrichment between the CK group, RY group, and SY group from the KEGG database through enrichment analysis and topology analysis. In the CK vs RY group, KEGG is mainly enriched in Furfural degradation, Phenoline metabolism, Tropane, piperidine and pyridine alkaloid biosynthenosis, Nucleotide metabolism, Purine metabolism (Fig 6A); The CK vs SY group KEGG is mainly enriched in Tropane, piperidine, and pyridine alkaloid biosynthenosis (Fig 6B); The KEGG in the RY vs SY group is mainly enriched in Cysteine and methyl metabololism, Glycine, serine and threonine metabololism, Aminoacyl Trna biosynthesis, Biosynthesis of variant plant secondary metabololites, Biosynthesis of amino acids, Arginine and proline metabololism, Biosynthesis of cofactors (Fig 6C) CK vs RY and CK vs SY share a common pathway called Tropane, piperidine, and pyridine alkaloid biosynthesis, therefore, this pathway may be a key weed control mechanism in fomesafen. The KEGG enrichment differential pathway of RY vs SY is the most abundant, therefore, there is the greatest mechanism difference between the RY group and the SY group. Other methods need to be used to remove resistant weeds, and compared to the CK vs SY group, the CK vs RY group has more mechanism differences.

### 3.3 Association analysis of transcriptome and metabolome

Used correlation analysis to study the potential regulatory network between DEG and DEM. For CK vs RY, CK vs SY, and RY vs SY groups, multiple DEMs are regulated by multiple DEGs, or a single DEG regulates multiple DEMs (S1–S3 Tables). This indicates that the action of fomesafen on *Amaranthus retroflexus L*. is a multi-gene and multi-metabolite interaction mechanism.

## 4. Discussion

Resistant weeds have always been a key factor affecting soybean yield. If the weed control effect is not good, it will lead to a serious reduction in soybean production or even crop failure. At present, the resistance of soybean weeds to herbicides is becoming increasingly severe in Heilongjiang Province, China, and *Amaranthus retroflexus L*. is one of them. Therefore, elucidating the resistance mechanism of *Amaranthus retroflexus L*. to fomesafen provides a theoretical basis for precise control of weeds in soybean fields, which has important theoretical and practical significance. Transcriptome technology has been widely applied in research fields such as gene and transcript recognition and mining, gene quantitative expression, etc. For example, RNA sequencing technology has been used to identify many genes involved in flavonoid biosynthesis in lettuce [22] and Dendrobium officinale [23]. At present, there are no reports on the transcriptional mechanisms of genes related to the sensitivity and resistance of *Amaranthus retroflexus L*. to flumexafen, only some reports on target genes. This study comprehensively investigated the transcriptional level changes after the action of fomesafen on *Amaranthus retroflexus L*. through transcriptome sequencing combined with statistical analysis. Differential expression analysis showed that the CK and RY groups detected the lowest number of DEGs. In addition, there is a significant overlap of DEGs between the RY and SY groups, as well as between the CK and SY groups, with overlapping genes accounting for more than 70% of the entire DEG. This indicates that resistant varieties of *Amaranthus retroflexus L*. have a significant resistance effect to fomesafen. There are research reports that after treatment with fomesafen, the relevant indicators of the same resistant population of the same *Amaranthus retroflexus L*. leaf age were less affected than those of the sensitive population, and the recovery rate was faster [21]. We validated this at the transcriptome level. The transcriptome results showed that compared with the CK group, the RY group had the least significantly different expression genes and was least affected by the SY group. Therefore, the phenotypic differences between the RY and SY groups may stem from changes in biogenetics, as indicated by transcriptome data.

Interestingly, we have also observed similar trend changes at the metabolomics level. Metabolomics analysis techniques have been widely used to detect and evaluate changes in metabolic products during plant growth and development stages [24,25]. The results of differential analysis of metabolites showed that CK and RY had the smallest difference in metabolites, and the RY group was closer to the CK group, which confirms the results of transcriptome analysis. At the same time, many DEMs are involved in the biosynthetic pathways of resistance and sensitivity in *Amaranthus retroflexus L*., indicating that the resistance and sensitivity of *Amaranthus retroflexus L*. are a multi-network interaction mechanism.

Previous studies have found that the structure and quantity of leaf tissue, palisade tissue, and organs in the sensitive branching group of *Amaranthus retroflexus L*. under electron microscopy were less affected than those in the SY group after treatment with fomesafen on stems and leaves, and the recovery ability was strong. Stomata still had the function of opening and closing; The combination therapy of P450 inhibitors and fomesafen can significantly increase the sensitivity of the RY group, and the development of drug resistance may be related to the enhancement of P450 activity; After application, the activities of the metabolic enzyme GST and target enzyme PPO in the resistant RY group were not significantly inhibited, but could recover after a period of time [26]. The above results indicate that the resistance mechanism of the RY group to fomesafen may be related to the recovery ability after tissue damage, the opening and closing function of stomata after application, the activity of degradation metabolic enzymes GST and P450s, and the target enzyme PPO. Therefore, we detected three genes associated with P450 GST and other enzyme systems such as POD, PPO, CAT and SOD through fluorescence quantitative PCR. We found significant changes in the expression of the two genes compared to the CK and SY groups. Therefore, our research results confirm that P450 GST, POD, PPO, CAT, and SOD are involved in the occurrence and development mechanism of resistant *Amaranthus retroflexus L.*. GST is related to the resistance of *Amaranthus retroflexus L.*. Glutathione is a short peptide formed by intermolecular dehydration of glutamic acid, cysteine and glycine, and is a tripeptide. The KEGG in the RY vs SY group is mainly enriched in Cysteine and metal metabololism, Glycine, serine and threonine metabololism, Aminoacyl-trna biosynthesis, biosynthesis of variant plant secondary metabololites, biosynthesis of amino acids, arginine and proline metabololism, Biosynthesis of cofactors. Therefore, the resistance mechanism of *Amaranthus retroflexus L*. may mainly be generated by the metabolic pathway mechanism of amino acids.

## 5. Conclusion

This study is the first to investigate the resistance pathways of *Amaranthus retroflexus L*. to fomesafen based on transcriptome and metabolomics techniques. Compared to the sensitive population, the resistant population showed significant differences in gene and metabolite expression, suggesting genetic differences between the resistant and sensitive populations. This study comprehensively explored the expression and pathways differences of resistant populations at the transcriptional and metabolomic levels for the first time, providing a theoretical basis for further in-depth research on the mechanisms of resistant populations and targeted prevention and control strategies.

## Supporting information

S1 Table Correlation analysis of differential genes and metabolites between CK groups and RY groups. (XLS)

S2 Table Correlation analysis of differential genes and metabolites between CK groups and SY groups. (XLS)

S3 Table Correlation analysis of differential genes and metabolites between SY groups and RY groups. (XLS)
